# Association of physical activity with sarcopenia evaluated based on muscle mass and strength in older adults: 2008–2011 and 2014 − 2018 Korea National Health and Nutrition Examination Surveys

**DOI:** 10.1186/s12877-022-02900-3

**Published:** 2022-03-17

**Authors:** Je Hyun Seo, Young Lee

**Affiliations:** Veterans Health Service Medical Center, Veterans Medical Research Institute, Jinhwangdo-ro 61-gil 53, Gangdong-gu Seoul, Korea

**Keywords:** Aging, Muscle mass, Sarcopenia, Muscle strength, Physical activity

## Abstract

**Background:**

Adequate physical activity (PA) is essential for preventing sarcopenia in older adults. However, there are insufficient epidemiological data on the intensity of PA needed to prevent age-related sarcopenia. The purpose of this study was to investigate the association of PA intensity with skeletal muscle mass and muscle strength.

**Methods:**

This was a population-based study with a cross-sectional design that was conducted using data from the 2008 − 2011 and 2014 − 2018 Korea National Health and Nutrition Examination Surveys, which included a total of 11,162 participants aged ≥ 60 years. PA was assessed using the results of a questionnaire and organized by intensity, frequency, and duration. The study population was divided into the following groups based on PA intensity: no exercise, walking only, moderate PA, and vigorous PA. To assess sarcopenia, skeletal muscle index (SMI) and hand grip strength (HGS) were measured as indicators of muscle mass and strength, respectively. Logistic regression analysis was used to explore the relationship between PA intensity and sarcopenia.

**Results:**

SMI and HGS were significantly higher in men and women engaged in moderate to vigorous PA than in those who did not exercise. The odds ratios (ORs) for sarcopenia defined based on SMI and HGS were lowest in men engaged in vigorous PA (0.444, 95% confidence interval [CI]: 0.242 − 0.818 and 0.450, 95% CI: 0.228 − 0.890, respectively). In women, the OR for sarcopenia defined based on HGS was the lowest in the group engaged in vigorous PA (0.441, 95% CI: 0.199 − 0.975), while there was no risk reduction for sarcopenia defined based on SMI.

**Conclusions:**

Moderate to vigorous PA was highly correlated with SMI and HGS in men and women. Intensive PA was positively correlated with sarcopenia prevention, which can be monitored using HGS.

## Background

Sarcopenia, a progressive decrease in skeletal muscle mass and function, is associated with poor quality of life, disability, and mortality [1–3]. Sarcopenia has become a serious public health issue in Korea due to the steady increase in the proportion of Koreans aged 65 years or older [3]. Accordingly, nationwide research on sarcopenia is urgently needed. Sarcopenia is diagnosed based on assessments of muscle mass, muscle strength, and physical performance. However, the criteria for diagnosing sarcopenia are inconsistent [4–8], and various parameters, such as muscle mass, appendicular skeletal muscle mass (ASM), and lean muscle mass, have been used in different studies. The most recent and popular criteria, the consensus of the 2019 Asian Working Group for Sarcopenia (AWGS) [9], provided cut-off values for skeletal muscle index (SMI) and hand grip strength (HGS) as measures of muscle mass and strength, respectively.

Various factors such as mitochondrial oxidative stress, apoptosis, and mitophagy and proteins such as myostatin and inflammatory cytokines are involved in the pathogenesis of sarcopenia [4]. Regular physical activity (PA) is recommended as a safe strategy to counter the loss of muscle mass and strength that occurs with aging [10]. Physical activity in the form of aerobic exercise (cycling, dancing, sports), resistance exercise (squats, weightlifting), and a combination of the two have been shown to prevent muscle atrophy and produce beneficial preventive and therapeutic effects via various mechanisms [11–14]. Although PA may indirectly affect other health parameters, it is an important factor associated with muscle strength and mass [15]. A previous meta-analysis on the relationship between sarcopenia and PA in older individuals demonstrated that PA reduces the odds of acquiring sarcopenia in later life (odds ratio [OR] = 0.45; 95% confidence interval [CI]: 0.37 − 0.55) [16]. A subsequent meta-analysis on the effects of nutrition and PA on sarcopenia revealed that PA positively impacted muscle mass and function in healthy participants, with limited effects of nutritional supplements [17].

In the Korea National Health and Nutrition Examination Survey (KNHANES), muscle mass and strength data were measured in different phases (2008 − 2011 for muscle mass data and 2014 − 2018 for muscle strength data). Hence, analyzing these data may provide novel insights into the association between PA and muscle mass and strength. Although numerous studies on the association between sarcopenia and factors such as nutrition and metabolic disease have been performed using KNHANES data [18–20], there is a paucity of studies analyzing the relationship between sarcopenia and PA using the two axes of muscle mass and strength. This could be due to the difficulty of statistical analysis of PA classification and quantification, extensive analysis required for data from different phases, and multiple factors related to the KNHANES. Recently, our study group conducted studies to analyze osteoporosis [21] and metabolic syndrome [22] using KNHANES data by classifying PA according to intensity, frequency, and duration. These studies highlighted the feasibility of analyzing the association between metabolic conditions and PA. Therefore, the purpose of this study was to investigate the relationship between PA amount, which includes intensity, frequency, and duration, and muscle mass and strength in older adults using data from the 2008–2011 and 2014 − 2018 KNHANES.

## Methods

### Study design and participants

This study used data from the KNHANES datasets from 2008–2011 and 2014 − 2018 produced by the Korea Disease Control and Prevention Agency. KNHANES is a nationwide survey with a cross-sectional design used to evaluate the health and nutritional status of the Korean population through medical history taking, physical examinations, health behavior surveys, and anthropometric and biochemical measurements. The Institutional Review Board of the VHS Medical Center approved the study protocol and waived the requirement for informed consent (IRB No. 2021–05-006) due to the retrospective nature of the study. The study was conducted in compliance with the Declaration of Helsinki.

**Fig. 1:**
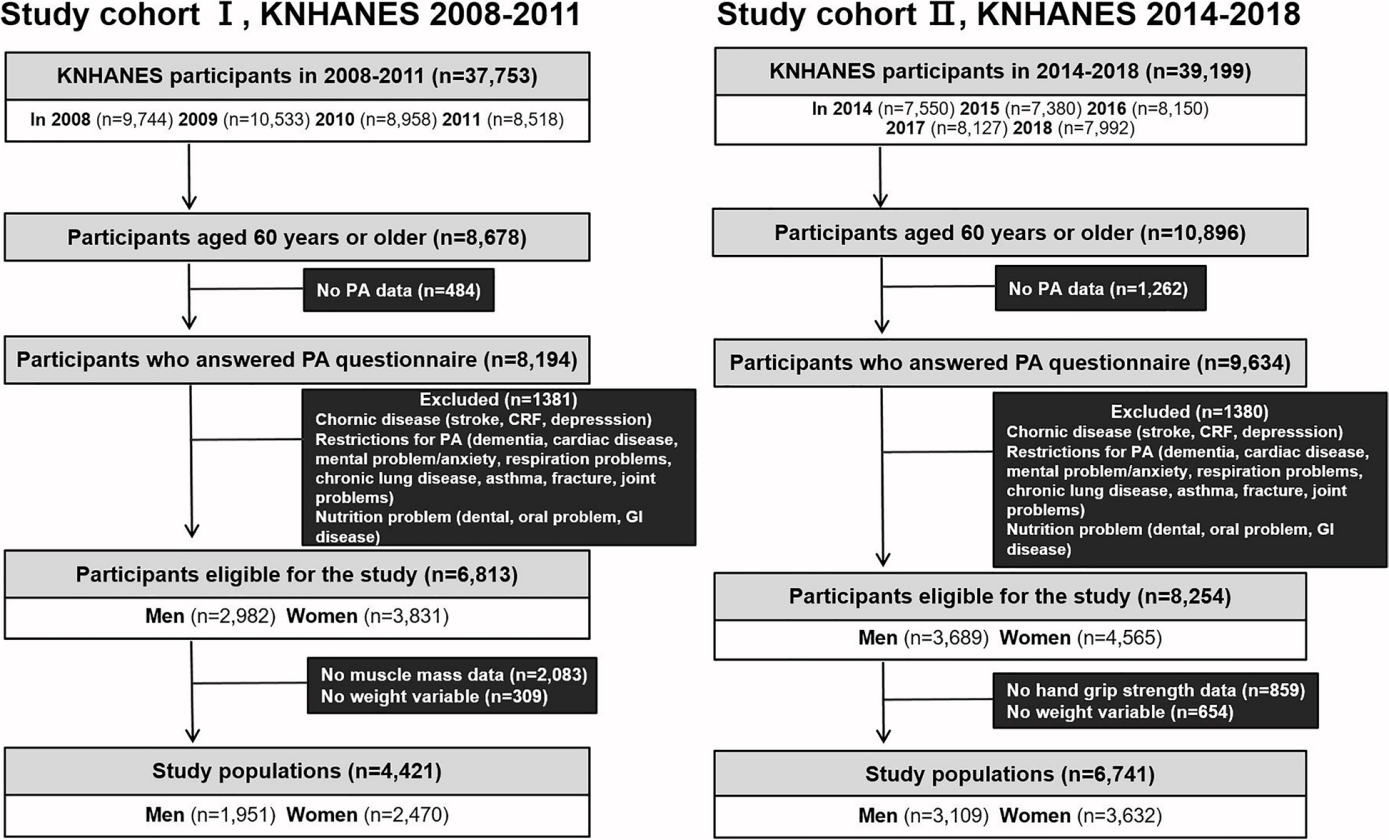
Flowchart of participant enrolment according to cohort. The KNHANES 2008 − 2011 and KNHANES 2014 − 2018 obtained data on muscle mass and hand grip strength, respectively. KNHANES, Korea National Health and Nutrition Examination Survey; PA, physical activity

We analyzed data of 8,678 participants aged 60 years or older from the 2008–2011 KNHANES and 10,896 participants aged 60 years or older from the 2014 − 2018 KNHANES (Fig. 1). The exclusion criteria were as follows: missing PA data (*n* = 484 and *n* = 1,262, respectively) and conditions that affect muscle condition for exclusion of secondary sarcopenia, including chronic disease, restriction of PA, and/or nutritional issues (*n* = 1,381 and *n* = 1,380, respectively). In total, 6,813 participants (2,982 men and 3,831 women) from the 2008–2011 KNHANES and 8,254 participants (3,689 men and 4,565 women) from the 2014 − 2018 KNHANES were eligible for participation in this study. Participants with missing data on muscle parameters (*n* = 2,083 for muscle mass data, *n* = 859 for HGS data) and weight variables (*n* = 309 and *n* = 654, respectively) were excluded from the analysis. A final total of 4,421 participants (1,951 men and 2,470 women) from the 2008–2011 KNHANES and 6,741 participants (3,109 men and 3,632 women) from the 2014–2018 KNHANES were included in the analyses.

### Assessments of skeletal muscle mass and strength

Whole and regional body compositions were measured with dual-energy X-ray absorptiometry (DXA) (QDR4500A; Hologic Inc., Bedford, MA). In the 2008–2011 KNHANES, ASM was calculated as the sum of the mass of the skeletal muscles in the arms and legs measured with DXA, under the assumption that all non-fat and non-bone tissues were skeletal muscles. SMI was calculated by dividing the ASM by height squared (ASM/height2), and this value was used as an indicator of skeletal muscle mass. In the 2014 − 2018 KNHANES, muscle strength was measured as HGS using a digital hand dynamometer (T.K.K 5401, Takei, Tokyo, Japan). With the participant in a standing position and forearm extended in a sideways position away from the body at the thigh level, participants were instructed to exert maximum grip strength three times each with the left and right hands, and the findings for the dominant hand were recorded. A rest interval of at least 30 s was allowed between each measurement. The participants were instructed to squeeze the dynamometer continuously with full force for at least 3 s. The average of the three trials for each hand was recorded. Based on the consensus of the 2019 AWGS [9], low muscle mass for sarcopenia was defined as an SMI < 7.0 kg/m2 for men and < 5.4 kg/m2 for women, and low muscle strength was defined as HGS < 28 kg for men and < 18 kg for women.

### Assessment of physical activity: Intensity, frequency, and duration

The International Physical Activity Questionnaire-Short Form (IPAQ-SF) was used to assess PA. The questionnaires were used to determine the intensity, frequency, and duration of PA performed by the participants, who were then grouped according to our previously reported classification system [21, 22]. In brief, participants were questioned as to whether they had engaged in different types of PA for exercise for at least 10 min over the past week. PAs were categorized as walking only, moderate PA, and vigorous PA, which is the classification system used in our previous studies [21, 22].

### Statistical analyses

All analyses were performed using the sample weights from the KNHANES data. When characterizing the participants according to PA intensity, data were expressed as means with standard error (SE) for continuous variables and percentages with SE for categorical variables. Continuous variables were analyzed using the independent t-test or analysis of variance, while categorical variables were analyzed using the Rao–Scott chi-square test. Age, body mass index (BMI), total energy intake, total protein intake, and total fat intake were considered continuous independent variables, whereas, smoking, alcohol intake, monthly household income, education level, diabetes, PA intensity, PA frequency, and PA duration were considered categorical independent variables. Subgroups were compared by applying the post-hoc Bonferroni correction after the t-test.

The following logistic regression models for sarcopenia were sequentially applied: unadjusted; model 1: adjusted for age; model 2: adjusted for age, smoking, drinking, alcohol intake, monthly household income, total energy intake, total protein intake, total fat intake, education level, and diabetes; model 3: adjusted for age and BMI; and model 4: adjusted for age, BMI, smoking, drinking, alcohol intake, monthly household income, total energy intake, total protein intake, total fat intake, education level, and diabetes. In addition, linear regression analysis was performed in model 4 to analyze the trends in SMI or HGS according to PA intensity, frequency, and duration in each PA group. Since adjustment for variables was performed, linear regression analysis was considered more suitable for trend analysis than analysis of variance. Statistical analyses were performed using the R 3.6.3 program (R Foundation, Vienna, Austria) and statistical significance was set at *P* < 0.05.

## Results

### Characteristics of the study participants

#### 2008 − 2011 KNHANES: study cohort I

In both men and women, the mean age was lower in the vigorous PA group than in the no exercise group (all *P* < 0.001, Table 1). In addition, BMI differed by PA group in men (*P* = 0.012) but not in women (*P* = 0.958). The proportion of sarcopenia defined based on SMI in all participants was 36.5% for men and 20.8% for women. The no exercise group exhibited the highest sarcopenia ratio (48.9% for men and 23.4% for women). No significant difference in smoking status was observed (*P* = 0.248), but alcohol consumption and monthly income significantly differed according to PA group in men (*P* = 0.036 and *P* < 0.001, respectively). Total energy intake was higher in the vigorous PA group than in the no exercise group, with a trend of marginal significance after adjustment for age in both men and women (*P* = 0.086 and *P* = 0.081, respectively). Total protein intake was higher in the vigorous exercise group than in the no exercise group after adjustment for age in men (*P* = 0.008), but no significant difference was observed in women (*P* = 0.110). Total fat intake did not differ according to age in the PA groups. The incidence of comorbidities such as hypertension, diabetes, and arthritis did not differ between men and women. A disparity in the duration and frequency of PA was identified between groups according to PA intensity in men, while the frequency of PA was significantly different in the moderate activity group than in the other groups in women.

**Table 1:** Baseline characteristics of study subjects of KNHANES 2008–2011 (Study cohort I)

	**Men**						**Women**					
	**Total**	**no Exercise**	**Walking-only**	**Moderate PA**	**Vigorous PA**		**Total**	**no Exercise**	**Walking-only**	**Moderate PA**	**Vigorous PA**	
	**(*n* = 1,951)**	**(*n* = 184)**	**(*n* = 866)**	**(*n* = 426)**	**(*n* = 475)**	***P***	**(*n* = 2,470)**	**(*n* = 420)**	**(*n* = 1,075)**	**(*n* = 567)**	**(*n* = 408)**	***P***
Age, years	68.19 ± 0.167	69.18 ± 0.559	68.85 ± 0.236	68.38 ± 0.339	66.49 ± 0.307	< 0.001	69.73 ± 0.174	72.59 ± 0.382	69.7 ± 0.241	69.24 ± 0.329	67.45 ± 0.382	< 0.001
		a	a	a	b			a	b	b	c	
BMI, kg/m^2^	23.44 ± 0.094	23.02 ± 0.233	23.29 ± 0.124	23.42 ± 0.198	23.91 ± 0.179	0.012	24.16 ± 0.083	24.12 ± 0.203	24.14 ± 0.13	24.24 ± 0.157	24.15 ± 0.182	0.958
		a	a	ab	b							
ASM, kg	20.07 ± 0.09	19.08 ± 0.217	19.85 ± 0.124	20.06 ± 0.161	20.83 ± 0.161	< 0.001	13.66 ± 0.052	13.27 ± 0.103	13.55 ± 0.077	13.91 ± 0.092	14.06 ± 0.11	< 0.001
		a	b	b	c			a	a	b	b	
age adjusted		19.15 ± 0.198	19.87 ± 0.118	20 ± 0.158	20.49 ± 0.161	< 0.001		13.59 ± 0.103	13.58 ± 0.072	13.9 ± 0.091	13.87 ± 0.106	0.014
age and BMI adjusted		19.32 ± 0.158	19.91 ± 0.106	20 ± 0.127	20.31 ± 0.12	< 0.001		13.53 ± 0.09	13.58 ± 0.061	13.88 ± 0.085	13.9 ± 0.1	0.001
SMI, kg/m2	7.29 ± 0.028	7.06 ± 0.067	7.21 ± 0.035	7.33 ± 0.05	7.5 ± 0.047	< 0.001	5.93 ± 0.018	5.86 ± 0.038	5.89 ± 0.026	6 ± 0.033	6.01 ± 0.037	0.001
		a	ab	b	c			a	a	b	b	
age adjusted		7.08 ± 0.063	7.21 ± 0.034	7.32 ± 0.048	7.41 ± 0.047	< 0.001		5.91 ± 0.039	5.89 ± 0.025	5.99 ± 0.033	5.98 ± 0.038	0.036
age and BMI adjusted		7.14 ± 0.047	7.23 ± 0.026	7.32 ± 0.035	7.35 ± 0.035	< 0.001		5.88 ± 0.034	5.89 ± 0.019	5.98 ± 0.03	6 ± 0.034	0.002
Sarcopenia, %	36.5 (1.44)	48.9 (4.53)	40.3 (1.97)	34.1 (2.95)	27.2 (2.58)	< 0.001	20.8 (1.11)	23.4 (2.62)	23.8 (1.63)	16.1 (2.12)	15.3 (2.15)	0.002
Alcohol consumption, %						0.036						0.090
None	26.3 (1.24)	33 (4.2)	28.8 (1.83)	26.1 (2.75)	19.5 (2.11)		61.2 (1.23)	64.4 (2.91)	59.9 (1.84)	65.3 (2.41)	56.2 (2.98)	
Moderate	34.8 (1.5)	30.1 (4.76)	34 (2.06)	33.5 (2.98)	38.8 (2.62)		33.9 (1.18)	31 (2.84)	35.9 (1.76)	29.7 (2.26)	36.6 (2.76)	
Heavy	38.9 (1.45)	36.9 (4.05)	37.1 (1.96)	40.4 (3.14)	41.7 (2.65)		4.9 (0.48)	4.6 (1.27)	4.2 (0.75)	5 (1.02)	7.2 (1.31)	
Smoking status, %						0.248						0.163
Never	15.1 (0.96)	12.2 (2.53)	13.8 (1.33)	16.6 (2.04)	17.3 (2.04)		89.7 (0.78)	86.5 (2.18)	89.2 (1.24)	92 (1.42)	91.9 (1.75)	
Ex-	56.2 (1.38)	53.6 (4.33)	56.4 (2.14)	54.2 (2.87)	58.3 (2.68)		5.5 (0.64)	6.6 (1.73)	6.6 (1.06)	3.8 (1.08)	3.6 (1.1)	
Current	28.7 (1.28)	34.3 (4.19)	29.7 (2.07)	29.2 (2.62)	24.4 (2.37)		4.7 (0.54)	7 (1.59)	4.2 (0.76)	4.3 (0.99)	4.5 (1.4)	
Monthly household income, %						< 0.001						0.053
Lowest	35.7 (1.42)	42.3 (4.31)	41.2 (2.12)	33.3 (2.78)	25.5 (2.26)		47.2 (1.4)	54.6 (2.98)	46.7 (2.04)	46.6 (2.66)	41.8 (2.93)	
Medium-lowest	28.6 (1.24)	27.3 (3.89)	28.2 (1.92)	29.1 (2.68)	29.3 (2.34)		25.1 (1.07)	23.2 (2.47)	23.7 (1.52)	28.1 (2.45)	27.2 (2.73)	
Medium-highest	19.7 (1.03)	22.6 (3.76)	17.2 (1.44)	21.4 (2.53)	21.7 (2.17)		16 (0.98)	13.9 (2.13)	17.6 (1.49)	15 (1.98)	15.3 (2.18)	
Highest	16 (1.14)	7.8 (2.35)	13.4 (1.47)	16.2 (2.05)	23.5 (2.59)		11.7 (0.89)	8.3 (1.59)	12.1 (1.33)	10.3 (1.57)	15.7 (2.34)	
Education level, %						< 0.001						< 0.001
≤ Elementary school	43.4 (1.68)	63.3 (4.52)	42.8 (2.2)	48.8 (3.09)	33 (3.07)		80.7 (1.09)	89.7 (1.88)	81.1 (1.47)	82.9 (1.97)	67.1 (3.08)	
Middle school	21.3 (1.28)	19.1 (3.77)	23.9 (1.69)	19.7 (2.84)	18.7 (2.11)		8.9 (0.69)	6.2 (1.35)	8.5 (0.99)	8.7 (1.47)	13.4 (2.16)	
High school	22.5 (1.14)	13.4 (2.8)	22.1 (1.78)	18 (2.18)	30.3 (2.65)		7.9 (0.72)	3.7 (1.03)	7.6 (0.92)	6.2 (1.16)	15.7 (2.74)	
≥ College	12.7 (1.16)	4.3 (2.07)	11.2 (1.38)	13.5 (2.03)	18 (2.8)		2.4 (0.42)	0.3 (0.24)	2.7 (0.63)	2.2 (0.73)	3.9 (1.11)	
Total energy intake, kcal/d	2012.86 ± 21.097	1910.97 ± 67.628	1958.17 ± 29.755	2049.95 ± 39.798	2117.9 ± 39.757	0.002	1451.24 ± 13.886	1418.44 ± 30.895	1419.05 ± 20.332	1494.17 ± 27.948	1524.31 ± 26.831	0.004
		a	a	ab	b			a	a	ab	b	
age adjusted		1927.53 ± 63.721	1962.41 ± 28.82	2038.84 ± 36.744	2047.26 ± 38.99	0.086		1478.5 ± 30.702	1425.18 ± 19.26	1491.67 ± 27.285	1488.5 ± 28.532	0.081
Total protein intake, g/d	68.24 ± 0.976	61.57 ± 2.874	65.64 ± 1.379	69.28 ± 1.849	74.48 ± 1.862	< 0.001	46.58 ± 0.602	43.46 ± 1.162	45.55 ± 0.853	48.1 ± 1.257	50.9 ± 1.439	< 0.001
		a	a	ab	b			a	ab	bc	c	
age adjusted		62.26 ± 2.632	65.82 ± 1.365	68.82 ± 1.742	71.55 ± 1.812	0.008		46.13 ± 1.147	45.82 ± 0.831	47.99 ± 1.206	49.31 ± 1.497	0.110
Total fat intake, g/d	31.08 ± 0.721	28.28 ± 2.325	29.63 ± 1.015	30.38 ± 1.278	35.23 ± 1.826	0.041	18.79 ± 0.41	16.71 ± 0.673	18.48 ± 0.612	19.29 ± 0.776	21.24 ± 0.934	0.001
		ab	a	ab	b			a	ab	ab	b	
age adjusted		28.72 ± 2.201	29.75 ± 1.011	30.08 ± 1.225	33.35 ± 1.744	0.282		18.37 ± 0.678	18.65 ± 0.607	19.22 ± 0.743	20.25 ± 0.986	0.461
Hypertension, %	40.7 (1.34)	28.9 (3.93)	42.5 (2.02)	42.8 (3.42)	40.3 (2.64)	0.046	50 (1.31)	54.8 (2.8)	49.7 (2.05)	49.4 (2.88)	46.4 (3.01)	0.276
Diabetes, %	16.5 (1)	21.2 (3.9)	16.1 (1.42)	15.9 (2.18)	15.9 (2.14)	0.553	16 (0.96)	18.7 (2.55)	16 (1.45)	14.3 (1.91)	15.1 (2.26)	0.552
Arthritis, %	12.8 (0.88)	14.9 (3.12)	12 (1.29)	13.1 (2.23)	13.3 (1.81)	0.829	41.6 (1.19)	40.6 (3.13)	41.6 (1.84)	41.4 (2.76)	42.9 (2.66)	0.955
Exercise frequency, times/wk												
Walking activity	4.53 ± 0.075	0	5.32 ± 0.084	4.63 ± 0.179	4.75 ± 0.142	< 0.001	4.05 ± 0.08	0	4.89 ± 0.096	4.87 ± 0.126	4.8 ± 0.148	0.873
			a	b	b							
Moderate activity	1.41 ± 0.071	0	0	4.12 ± 0.139	2.3 ± 0.148	< 0.001	1.27 ± 0.06	0	0	4.01 ± 0.11	2.78 ± 0.163	< 0.001
Vigorous activity	0.91 ± 0.055	0	0	0	3.57 ± 0.129		0.58 ± 0.044	0	0	0	3.65 ± 0.131	
Exercise duration, min/wk												
Walking activity	430.6 ± 17.5	0	508.7 ± 27.3	393.66 ± 30.7	481.7 ± 28.2	0.014	275.03 ± 10.6	0	324.8 ± 15.8	308.3 ± 20.3	375.9 ± 26.9	0.103
			a	b	a				a	b	a	
Moderate activity	190 ± 15.3	0	0	581.2 ± 43.4	290.3 ± 30.9	< 0.001	154.21 ± 11.0	0	0	460.5 ± 33.3	371.5 ± 35.0	0.060
Vigorous activity	124.3 ± 10.5	0	0	0	489.72 ± 33.4		93.11 ± 9.4	0	0	0	587.1 ± 46.4	

Data with the same lowercase letters indicate non-specific differences between groups, while those with different letters are statistically different, based on post hoc test

Data are expressed as the mean ± SE or the percentage (SE)

#### 2014 − 2018 KNHANES: study cohort 

In both men and women, the mean age was lower in the vigorous PA group than in the no exercise group (all *P* < 0.001, Table 2). The proportion of sarcopenia defined based on HGS was 19.0% in men and 31.6% in women. The no exercise group had the highest sarcopenia ratio (24.6% in men and 50.5% in women). Alcohol consumption, smoking status, education level, and monthly income differed between men and women according to PA intensity (all *P* < 0.01). Total energy intake, total protein intake, and total fat intake in men and women were higher in the moderate PA and vigorous PA groups than in the no exercise group after adjustment for age (all *P* < 0.001). The incidence of comorbidities such as hypertension, diabetes, and arthritis did not differ in men among the PA intensity groups. For women, the incidence of hypertension and diabetes was higher in the no exercise group than in the vigorous PA group (all *P* < 0.001). Discrepancies in the frequency and duration of PA were identified between the PA groups in men and women, except for the duration of PA in the walking-only group.

**Table 2:** Baseline characteristics of study subjects of KNHANES 2014–2018 (Study cohort II)

	**Men**						**Women**					
	**Total**	**no Exercise**	**Walking-only**	**Moderate PA**	**Vigorous PA**		**Total**	**no Exercise**	**Walking-only**	**Moderate**	**Vigorous**	
	**(*n* = 3,109)**	**(*n* = 595)**	**(*n* = 1,684)**	**(*n* = 603)**	**(*n* = 227)**	***P***	**(*n* = 3,632)**	**(*n* = 823)**	**(*n* = 2,243)**	**(*n* = 469)**	**(*n* = 97)**	***P***
Age, years	69.49 ± 0.129	70.21 ± 0.303	70.14 ± 0.182	68.43 ± 0.236	66.21 ± 0.448	< 0.001	69.35 ± 0.13	72.63 ± 0.292	68.96 ± 0.161	67.02 ± 0.288	64.52 ± 0.518	< 0.001
		a	a	b	c			a	b	c	d	
BMI, kg/m^2^	23.87 ± 0.057	23.68 ± 0.137	23.84 ± 0.081	23.97 ± 0.127	24.22 ± 0.2	0.148	24.42 ± 0.063	24.72 ± 0.156	24.42 ± 0.076	24.17 ± 0.161	23.39 ± 0.312	0.001
								a	a	ab	b	
Hand grip strength, right hand, kg	32.8 ± 0.158	31.48 ± 0.36	32.17 ± 0.198	34.2 ± 0.332	36.36 ± 0.467	< 0.001	19.59 ± 0.111	17.53 ± 0.236	19.84 ± 0.129	21.12 ± 0.234	22.08 ± 0.443	< 0.001
		a	a	b	c			a	b	c	c	
age adjusted		31.8 ± 0.312	32.45 ± 0.174	33.57 ± 0.306	34.52 ± 0.401	< 0.001		18.5 ± 0.211	19.6 ± 0.118	20.25 ± 0.217	20.38 ± 0.419	< 0.001
age and BMI adjusted		31.92 ± 0.303	32.46 ± 0.169	33.56 ± 0.303	34.48 ± 0.401	< 0.001		18.53 ± 0.204	19.63 ± 0.118	20.31 ± 0.219	20.56 ± 0.429	< 0.001
Hand grip strength, left hand, kg	31.84 ± 0.152	30.78 ± 0.36	31.19 ± 0.189	33.12 ± 0.318	35.32 ± 0.463	< 0.001	18.69 ± 0.108	16.97 ± 0.225	18.89 ± 0.126	20.05 ± 0.211	20.83 ± 0.434	< 0.001
		a	a	b	c			a	b	c	c	
age adjusted		31.07 ± 0.323	31.44 ± 0.168	32.54 ± 0.29	33.67 ± 0.446	< 0.001		17.88 ± 0.202	18.67 ± 0.115	19.23 ± 0.198	19.23 ± 0.423	< 0.001
age and BMI adjusted		31.21 ± 0.313	31.45 ± 0.163	32.54 ± 0.284	33.64 ± 0.446	< 0.001		17.92 ± 0.195	18.69 ± 0.115	19.28 ± 0.2	19.36 ± 0.428	< 0.001
Hand grip strength, Dominant hand, kg	33.69 ± 0.153	32.5 ± 0.356	33.03 ± 0.189	35 ± 0.32	37.39 ± 0.468	< 0.001	20.12 ± 0.11	18.13 ± 0.23	20.36 ± 0.128	21.62 ± 0.218	22.52 ± 0.425	< 0.001
		a	a	b	c			a	b	c	c	
age adjusted		32.81 ± 0.31	33.3 ± 0.165	34.39 ± 0.293	35.62 ± 0.428	< 0.001		19.1 ± 0.205	20.13 ± 0.116	20.75 ± 0.201	20.82 ± 0.407	< 0.001
age and BMI adjusted		32.94 ± 0.299	33.3 ± 0.161	34.39 ± 0.288	35.58 ± 0.428	< 0.001		19.13 ± 0.198	20.15 ± 0.116	20.81 ± 0.203	20.99 ± 0.414	< 0.001
Sarcopenia, %	19 (0.83)	24.6 (2.07)	21.2 (1.19)	13.5 (1.59)	5.2 (1.58)	< 0.001	31.6 (1.02)	50.5 (2.13)	28.6 (1.2)	20.1 (2.09)	13.5 (4.06)	< 0.001
Alcohol consumption, %						< 0.001						< 0.001
None	26.5 (0.97)	31.4 (2.31)	28 (1.3)	20.2 (1.81)	21.5 (3.17)		55.8 (1.07)	64.1 (2.16)	54.5 (1.37)	50.6 (2.43)	48.5 (5.83)	
Moderate	37.5 (1.1)	30.6 (2.28)	35.7 (1.41)	44.5 (2.23)	46.3 (3.7)		38.5 (1.03)	29.8 (2.04)	39.8 (1.32)	45.2 (2.42)	42.2 (5.7)	
Heavy	36 (1.02)	37.9 (2.39)	36.3 (1.36)	35.2 (2.2)	32.2 (3.5)		5.7 (0.43)	6.1 (1.05)	5.7 (0.53)	4.1 (1.05)	9.3 (3.03)	
Smoking status, %						< 0.001						0.003
Never	19.4 (0.83)	17.7 (1.85)	20.7 (1.16)	17.2 (1.87)	20 (2.84)		94.7 (0.5)	92.8 (1.18)	94.7 (0.61)	96.6 (0.86)	99 (1.01)	
Ex-	61.3 (1.02)	55.9 (2.3)	59.7 (1.45)	69 (2.24)	65.3 (3.7)		3.5 (0.36)	3.5 (0.79)	3.7 (0.48)	2.8 (0.8)	0 (0)	
Current	19.3 (0.83)	26.4 (2.21)	19.7 (1.17)	13.8 (1.65)	14.7 (2.95)		1.9 (0.35)	3.7 (0.91)	1.6 (0.38)	0.6 (0.32)	1 (1.01)	
Monthly household income, %						< 0.001						< 0.001
Lowest	30.4 (1.02)	39.2 (2.48)	33.1 (1.4)	22.2 (1.95)	14.2 (2.66)		39.8 (1.14)	55.5 (2.24)	38.1 (1.36)	27.6 (2.31)	16 (3.71)	
Medium-lowest	29.7 (0.91)	33.1 (2.19)	29.9 (1.34)	28.3 (1.95)	24.9 (3.13)		26.3 (0.84)	22.9 (1.7)	27.6 (1.06)	25.6 (2.32)	28.3 (5.08)	
Medium-highest	21.3 (0.83)	17.2 (1.74)	20.4 (1.17)	25 (2.03)	26.5 (3.41)		18.9 (0.83)	12.7 (1.44)	19.3 (1.03)	25.3 (2.52)	27.7 (5.4)	
Highest	18.6 (0.91)	10.5 (1.89)	16.6 (1.08)	24.5 (2.08)	34.5 (3.82)		14.9 (0.88)	8.9 (1.22)	15 (1.11)	21.5 (2.45)	28.1 (5.04)	
Education level, %						< 0.001						< 0.001
≤ Elementary school	34.8 (1.1)	51.4 (2.36)	36.7 (1.45)	22.7 (2.04)	16 (2.76)		60.5 (1.1)	80 (1.85)	58.7 (1.3)	42.7 (2.7)	38.7 (5.65)	
Middle school	17.3 (0.81)	18.9 (1.95)	17.9 (1.1)	15.1 (1.63)	15.1 (2.74)		15.4 (0.74)	8.3 (1.11)	16.5 (0.91)	20.2 (2.08)	17.9 (5.31)	
High school	27.1 (0.93)	20.7 (1.89)	26.4 (1.24)	30.9 (2.21)	36 (3.56)		16.2 (0.84)	8.8 (1.39)	17.4 (1.04)	20.2 (2.22)	26.7 (5.37)	
≥ College	20.8 (1.01)	8.9 (1.37)	19 (1.24)	31.3 (2.28)	32.9 (3.75)		7.9 (0.61)	2.9 (0.8)	7.4 (0.69)	16.9 (2.33)	16.7 (4.15)	
Total energy intake, kcal/d	2044.97 ± 17.355	1953.63 ± 39.179	1987.96 ± 22.029	2192.93 ± 37.591	2255.67 ± 67.919	< 0.001	1555.62 ± 13.166	1421.15 ± 25.589	1557.81 ± 16.407	1690.6 ± 34.464	1888.54 ± 86.786	< 0.001
		a	a	b	b			a	b	c	c	
age adjusted		1970.12 ± 39.446	2002.58 ± 21.396	2160.5 ± 36.994	2162.03 ± 71.302	< 0.001		1481.37 ± 26.143	1543.29 ± 16.058	1636.41 ± 34.057	1783.29 ± 84.939	< 0.001
Total protein intake, g/d	69.38 ± 0.723	63.4 ± 1.496	66.69 ± 0.924	77.33 ± 1.635	80.5 ± 2.869	< 0.001	51.51 ± 0.546	43.94 ± 1.017	51.86 ± 0.668	58.98 ± 1.502	65.4 ± 3.118	< 0.001
		a	a	b	b			a	b	c	c	
age adjusted		64.09 ± 1.496	67.31 ± 0.912	75.97 ± 1.619	76.57 ± 2.998	< 0.001		46.89 ± 1.038	51.15 ± 0.633	56.32 ± 1.441	60.23 ± 3.081	< 0.001
Total fat intake, g/d	34.38 ± 0.561	30.26 ± 1.046	32.41 ± 0.706	39.78 ± 1.358	43 ± 2.603	< 0.001	25.89 ± 0.405	20.75 ± 0.779	25.82 ± 0.479	32.03 ± 1.153	37.3 ± 2.552	< 0.001
		a	a	b	b			a	b	c	c	
age adjusted		30.72 ± 1.061	32.81 ± 0.7	38.89 ± 1.35	40.4 ± 2.668	< 0.001		22.86 ± 0.806	25.31 ± 0.457	30.13 ± 1.113	33.61 ± 2.557	< 0.001
Hypertension, %	48.5 (1.04)	51.5 (2.34)	49.6 (1.38)	45.6 (2.33)	42.7 (3.86)	0.107	49.1 (0.99)	57.2 (2.04)	48.9 (1.25)	39.7 (2.56)	35.1 (5.64)	< 0.001
Diabetes, %	20.2 (0.84)	22.8 (1.97)	19.8 (1.1)	20.8 (1.96)	16.3 (2.62)	0.274	16.8 (0.74)	20.2 (1.73)	17.1 (0.93)	11.2 (1.53)	10.3 (3.15)	0.001
Arthritis, %	11.8 (0.66)	12.6 (1.53)	11.9 (0.96)	11.1 (1.34)	11.3 (2.71)	0.914	39.7 (0.97)	43 (2.11)	38.7 (1.26)	40.9 (2.59)	33.5 (5.32)	0.192
Exercise frequency, times/wk												
Walking activity	3.94 ± 0.062	0	4.96 ± 0.06	4.44 ± 0.116	4.55 ± 0.182	< 0.001	3.71 ± 0.06	0	4.76 ± 0.056	4.29 ± 0.119	4.78 ± 0.302	0.002
			a	b	ab				a	b	ab	
Moderate activity	1.05 ± 0.043	0	0	4.16 ± 0.097	2.61 ± 0.187	< 0.001	0.57 ± 0.029	0	0	3.84 ± 0.097	2.38 ± 0.287	< 0.001
Vigorous activity	0.31 ± 0.026	0	0	0	3.72 ± 0.135		0.09 ± 0.011	0	0	0	3.58 ± 0.191	
Exercise duration, min/wk												
Walking activity	302.4 ± 9.4	0	377.3 ± 13.5	337.7 ± 16.8	378.57 ± 33.9	0.163	246.6 ± 7.8	0	315.9 ± 10.7	283.7 ± 18.6	332.9 ± 43.9	0.290
Moderate activity	78.5 ± 4.6	0	0	314.9 ± 17.3	189.2 ± 19.5	< 0.001	34.2 ± 2.8	0	0	228.7 ± 16.7	157.7 ± 30.2	0.043
Vigorous activity	22.81 ± 2.7	0	0	0	275.9 ± 25.7		4.71 ± 0.7	0	0	0	179.84 ± 19.99	

Data with the same lowercase letters indicate non-specific differences between groups, while those with different letters are statistically different, based on post hoc test

Data are expressed as the mean ± SE or the percentage (SE)

### Association between physical activity and skeletal muscle index

**Fig. 2:**
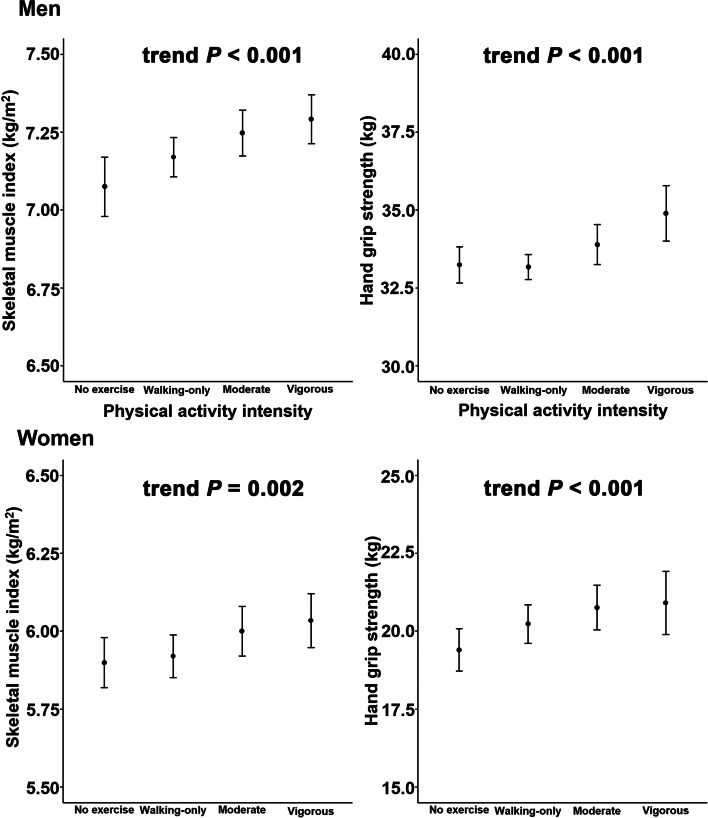
Mean skeletal muscle index and hand grip strength by physical activity intensity using trend analysis. Trend *P* using a linear regression model after adjusting for age, body mass index, smoking, drinking, monthly income, total energy intake, total protein intake, total fat intake, education level, and diabetes. Error bars indicate 95% confidence intervals

**Fig. 3:**
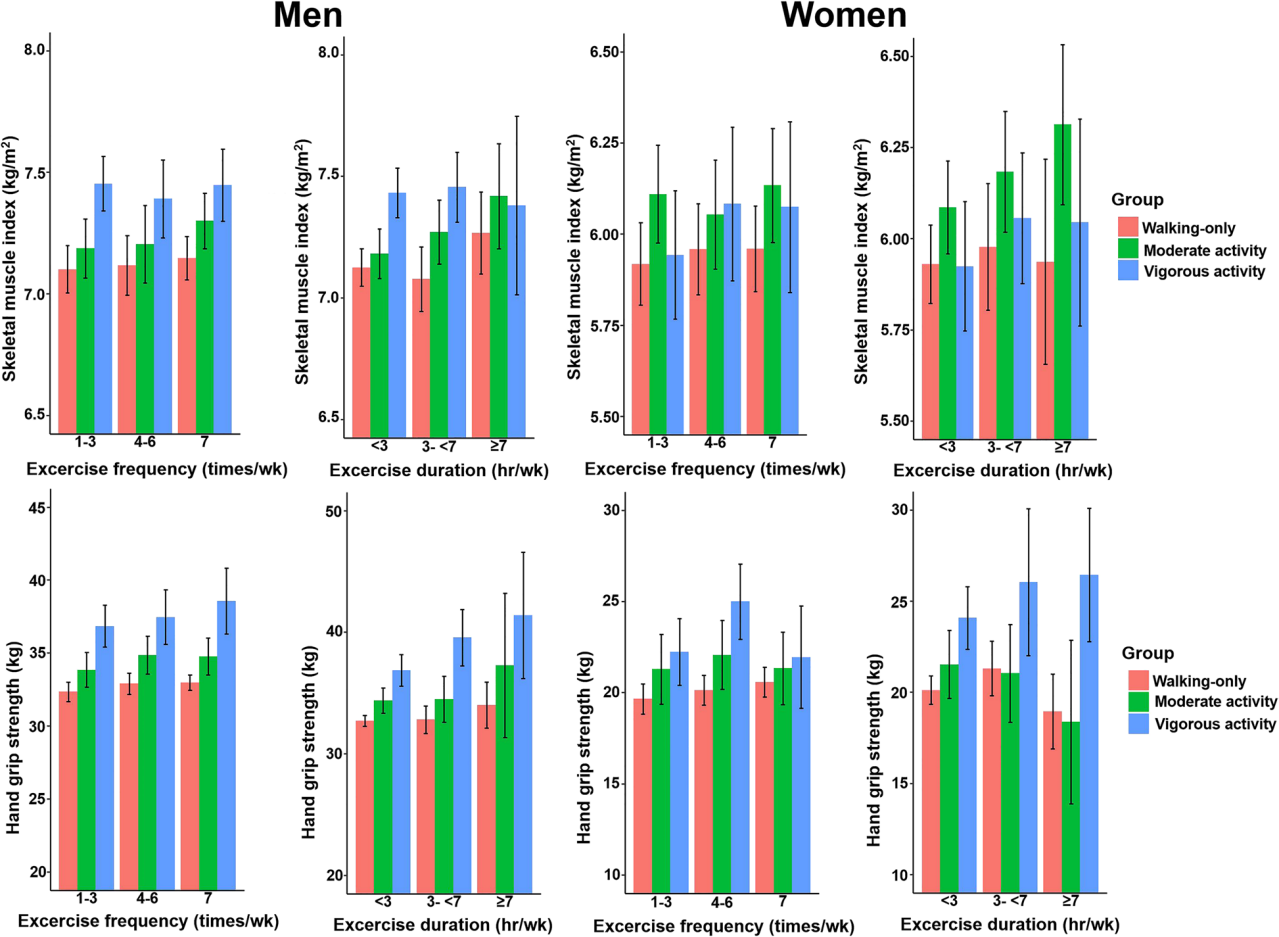
Mean skeletal muscle index and hand grip strength by physical activity frequency and duration. Linear regression model after adjusting for age, body mass index, smoking, drinking, monthly income, total energy intake, total protein intake, total fat intake, education level, and diabetes. Error bars indicate 95% confidence intervals

In men and women, both ASM and SMI increased according to PA intensity, and these differences existed even in the models adjusted for age and BMI (all *P* < 0.05, Table 1). Trend analysis showed that PA intensity was associated with SMI in women and men (*P* = 0.002 and *P* < 0.001, respectively; Fig. 2). In men, SMI values based on the frequency and duration of PA did not significantly differ according to PA intensity, except for the duration of PA in the walking-only group (*P* = 0.013, Table 3 and Fig. 3). In women, SMI values based on the frequency and duration of PA did not differ according to PA intensity, except in the walking-only group (*P* = 0.001) and the vigorous PA group, which showed significant differences according to exercise duration (*P* = 0.027).

**Table 3:** Adjusted mean values of skeletal muscle index and hand grip strength according to the frequency or duration of physical activities in men and women

	Men		Women	
	Skeletal muscle index	Hand grip strength	Skeletal muscle index	Hand grip strength
Walking-only group	*n* = 866	*n* = 1684	*n* = 1075	*n* = 2243
Frequency				
1-3	7.1 ± 0.05	32.35 ± 0.343	5.92 ± 0.058	19.65 ± 0.424
4-6	7.12 ± 0.062	32.9 ± 0.372	5.96 ± 0.064	20.13 ± 0.417
everyday	7.15 ± 0.045	32.97 ± 0.272	5.96 ± 0.06	20.57 ± 0.418
* P*	0.639	0.237	0.553	0.001
Duration				
<3	7.03 ± 0.048	5.93 ± 0.057	32.08 ± 0.32	19.82 ± 0.414
3- <7	7.14 ± 0.052	5.93 ± 0.065	32.96 ± 0.345	20.43 ± 0.419
≥7	7.18 ± 0.046	5.95 ± 0.061	33.36 ± 0.28	20.53 ± 0.441
* P*	0.013	0.878	0.001	0.008
Moderate PA group	*n* = 426	*n* = 603	*n* = 567	*n* = 469
Frequency				
1-3	7.19 ± 0.062	33.85 ± 0.605	6.11 ± 0.068	21.29 ± 0.976
4-6	7.21 ± 0.081	34.87 ± 0.66	6.05 ± 0.076	22.07 ± 0.969
everyday	7.3 ± 0.058	34.78 ± 0.645	6.13 ± 0.08	21.33 ± 1.014
* P*	0.273	0.218	0.488	0.152
Duration				
<3	7.16 ± 0.073	6.06 ± 0.07	33.85 ± 0.577	21.01 ± 0.916
3- <7	7.26 ± 0.058	6.07 ± 0.072	34.46 ± 0.729	21.96 ± 0.961
≥7	7.24 ± 0.061	6.2 ± 0.082	35.57 ± 0.725	22.04 ± 1.025
* P*	0.424	0.072	0.073	0.030
Vigorous PA group	*n* = 475	*n* = 227	*n* = 408	*n* = 97
Frequency				
1-3	7.45 ± 0.057	36.84 ± 0.73	5.94 ± 0.09	22.23 ± 0.932
4-6	7.39 ± 0.082	37.47 ± 0.947	6.08 ± 0.108	24.99 ± 1.051
everyday	7.45 ± 0.076	38.55 ± 1.153	6.07 ± 0.119	21.94 ± 1.429
* P*	0.748	0.369	0.058	0.011
Duration				
<3	7.41 ± 0.076	5.76 ± 0.09	36.54 ± 0.688	24.15 ± 0.851
3- <7	7.43 ± 0.058	6.06 ± 0.096	37.1 ± 0.853	23.00 ± 1.248
≥7	7.47 ± 0.07	6.08 ± 0.095	39.57 ± 1.124	24.69 ± 1.389
* P*	0.792	<0.001	0.027	0.404

Linear regression analysis adjusted with age, BMI, smoking, alcohol intake, total energy intake, total protein intake, total fat intake, monthly household income, education level and diabetes

### Association between physical activity and hand grip strength

HGS of the right, left, and dominant hands increased according to PA intensity, and these differences persisted after adjustment for age and BMI (all *P* < 0.001, Table 2). Trend analysis revealed that PA intensity was associated with HGS in both men and women (*P* < 0.001 and *P* < 0.001, respectively; Fig. 2). In men, HGS values based on the frequency and duration of PA did not significantly differ according to PA intensity, except for the duration of PA in the vigorous PA group (*P* < 0.001, Table 3 and Fig. 3). In women, the frequencies of PA based on HGS were significantly different in the walking-only and vigorous PA groups (*P* = 0.001, *P* = 0.011, respectively), while the PA duration as associated with HGS differed in the walking-only and moderate PA groups (*P* = 0.008, *P* = 0.030, respectively).

### Logistic regression model for physical activity

For men engaged in vigorous PA, the ORs of sarcopenia as defined based on SMI were 0.468 (95% CI: 0.298 − 0.734) in model 1, 0.529 (95% CI: 0.326 − 0.858) in model 2, 0.450 (95% CI: 0.250 − 0.808) in model 3, and 0.444 (95% CI: 0.242 − 0.818) in model 4 (Table 4). Men engaged in moderate PA also exhibited a lower risk of sarcopenia as defined based on SMI (OR = 0.559, 95% CI: 0.354 − 0.883 in model 1; OR = 0.606, 95% CI: 0.374 − 0.984 in model 2; OR = 0.505, 95% CI: 0.287 − 0.888 in model 3; and OR = 0.512, 95% CI: 0.289 − 0.907 in model 4); however, in women, there was no risk reduction for sarcopenia as defined based on SMI according to PA intensity.

**Table 4:** Odds ratio for sarcopenia according to physical activities intensity

	**no Exercise**	**Walking-only**	**Moderate PA**	**Vigorous PA**	***P***
**Men**					
**Sarcopenia (Skeletal muscle index)**	***n* = 184**	***n* = 866**	***n* = 426**	***n* = 475**	
Unadjusted	1	0.706 (0.485–1.029)	0.542 (0.351–0.837)†	0.390 (0.254–0.601)†	< 0.001
Model 1	1	0.710 (0.479–1.052)	0.559 (0.354–0.883)*	0.468 (0.298–0.734)†	< 0.001
Model 2	1	0.748 (0.485–1.152)	0.606 (0.374–0.984)*	0.529 (0.326–0.858)*	0.003
Model 3	1	0.671 (0.419–1.075)	0.505 (0.287–0.888)*	0.450 (0.250–0.808)†	0.005
Model 4	1	0.644 (0.395–1.049)	0.512 (0.289–0.907)*	0.444 (0.242–0.818)†	0.010
**Sarcopenia (Hand grip strength)**	***n* = 595**	***n* = 1684**	***n* = 603**	***n* = 227**	
Unadjusted	1	0.824 (0.636–1.068)	0.479 (0.337–0.680)†	0.166 (0.083–0.333)†	< 0.001
Model 1	1	0.831 (0.624–1.107)	0.636 (0.438–0.924)*	0.283 (0.143–0.563)†	< 0.001
Model 2	1	0.957 (0.729–1.257)	0.865 (0.595–1.259)	0.431 (0.216–0.861)*	0.040
Model 3	1	0.865 (0.646–1.158)	0.672 (0.460–0.982)*	0.293 (0.149–0.576)†	< 0.001
Model 4	1	0.996 (0.752–1.319)	0.912 (0.624–1.332)	0.450 (0.228–0.890)*	0.070
**Women**					
**Sarcopenia (Skeletal muscle index)**	***n* = 420**	***n* = 1075**	***n* = 567**	***n* = 408**	
Unadjusted	1	1.021 (0.737–1.414)	0.630 (0.433–0.918)*	0.593 (0.388–0.904)*	< 0.001
Model 1	1	1.186 (0.848–1.658)	0.745 (0.507–1.095)	0.770 (0.499–1.187)	0.018
Model 2	1	1.216 (0.858–1.724)	0.785 (0.526–1.172)	0.795 (0.508–1.245)	0.034
Model 3	1	1.213 (0.814–1.809)	0.726 (0.470–1.122)	0.649 (0.390–1.082)	0.005
Model 4	1	1.304 (0.861–1.975)	0.791 (0.510–1.227)	0.694 (0.413–1.164)	0.009
**Sarcopenia (Hand grip strength)**	***n* = 823**	***n* = 2243**	***n* = 469**	***n* = 97**	
Unadjusted	1	0.393 (0.327–0.472)†	0.246 (0.181–0.336)†	0.153 (0.076–0.307)†	< 0.001
Model 1	1	0.566 (0.466–0.689)†	0.446 (0.323–0.616)†	0.383 (0.183–0.800)*	< 0.001
Model 2	1	0.632 (0.516–0.775)†	0.541 (0.387–0.754)†	0.463 (0.210–1.021)	< 0.001
Model 3	1	0.566 (0.464–0.690)†	0.440 (0.318–0.608)†	0.368 (0.176–0.771)†	< 0.001
Model 4	1	0.628 (0.510–0.773)†	0.534 (0.382–0.747)†	0.441 (0.199–0.975)*	< 0.001

Unadjusted: no adjustment; model 1: adjusted by age; model 2: age, smoking, alcohol intake, total energy intake, total protein intake, total fat intake, monthly household income, education level and diabetes; model 3: age and BMI; model 4: age, BMI, smoking, alcohol intake, total energy intake, total protein intake, total fat intake, monthly household income, education level and diabetes

^*^: indicate, if *P* < 0.05, †: indicate, if *P* < 0.01

Men engaged in vigorous PA also showed a lower risk of sarcopenia as defined based on HGS (OR = 0.283, 95% CI: 0.143 − 0.563 in model 1; OR = 0.431, 95% CI: 0.216 − 0.861 in model 2; OR = 0.293, 95% CI: 0.149–0.576 in model 3; and OR = 0.450, 95% CI: 0.228 − 0.890 in model 4) (Table 4). For men engaged in moderate PA, the ORs of sarcopenia as defined based on HGS were significant only in models 1 (0.636, 95% CI: 0.438 − 0.924) and 3 (0.672, 95% CI: 0460 − 0.982). Women in the vigorous PA group also demonstrated a lower risk of sarcopenia as defined based on HGS (OR = 0.383, 95% CI: 0.183 − 0.800 in model 1; OR = 0.368, 95% CI: 0.176 − 0.771 in model 3; and OR = 0.441, 95% CI: 0.199 − 0.975 in model 4). For women in the moderate PA group, the ORs of sarcopenia as defined based on HGS were 0.446 (95% CI: 0.323 − 0.616) in model 1, 0.541 (95% CI: 0.387 − 0.754) in model 2, 0.440 (95% CI: 0.318 − 0.608) in model 3, and 0.534 (95% CI: 0.382 − 0.747) in model 4. In women, risk reduction was observed in those engaged in walking only, whereby ORs of sarcopenia as defined based on HGS were 0.566 (95% CI: 0.466 − 0.689) in model 1, 0.632 (95% CI: 0.516 − 0.775) in model 2, 0.566 (95% CI: 0.464 − 0.690) in model 3, and 0.628 (95% CI: 0.510 − 0.773) in model 4.

## Discussion

Our study showed a positive correlation between PA intensity and both SMI and HGS in men and women aged ≥ 60 years. Men engaged in moderate-to-vigorous PA had a lower risk of sarcopenia as defined based on SMI than in those who did not exercise, although this relationship was not observed in women. However, PA intensity was associated with a significant reduction in the risk of sarcopenia as defined based on HGS in both men and women.

It is well established that PA improves physical function and quality of life, thereby reducing the burden of chronic disease. Indeed, PA influences key drivers of aging, including chronic inflammation, oxidative damage, and reduced insulin-like growth factor signaling [23, 24]. Our results are similar to the results of a meta-analysis that recommended the use of regular vigorous intensity resistance training rather than walking alone to prevent sarcopenia in older adults [16]. Several studies have reported that resistance training mitigates sarcopenia via satellite cell proliferation and increases muscle hypertrophy [25, 26]. Although a decrease in daily PA due to the decline in muscle function with age is common, it remains unclear whether PA intensity can prevent muscle aging. In our study, PA intensity was associated with skeletal muscle mass, including SMI and ASM, which was consistent with a previous study on skeletal muscle mass in older women in Japan [27]. A recent study demonstrated that the risk of sarcopenic obesity due to active PA was decreased by 45% in men and 29% in women [28].

Muscle strength measurement is relatively simpler and less expensive than muscle mass measurement. HGS is a measure of muscle strength that is widely used for the evaluation of myopathy [29]. Poor HGS is independently associated with a high risk of falls in older adults [30]. A previous study on risk factors associated with low HGS using a similar cohort [31] reported that a low HGS was associated with various factors including alcohol consumption, exercise, education, and BMI. In our study, PA amount was classified according to its intensity, frequency, and duration, and cut-off values of HGS < 28 kg for men and < 18 kg for women were used according to the 2019 AWGS [9]. In contrast, in the previous study, the group engaged in 150 min or more of exercise was defined as a PA group, and cut-off values of 28.9 kg for men and 16.8 kg for women were used to define sarcopenia [31]. In our study, logistic regression analysis revealed a strong relationship between PA intensity and HGS, but the relationship between PA intensity and SMI did not exhibit a protective effect in women. This result differs from that of a recent study demonstrating that regular PA in older women promotes the maintenance of muscle mass and prevents sarcopenia [32]. This discrepancy could be due to 1) the definition for sarcopenia in women based on SMI being strict (20.8%) whereas that based on HGS is more conservative and sensitive (31.6%) in the AWGS criteria, and 2) differences in muscle mass and strength, which may be due to physiological differences between women and men, hormonal changes, and aging mechanisms [33]. This suggests that HGS in women more strongly reflects the effects of PA than SMI.

The International Exercise Recommendations in Older Adults (ICFSR) consensus guidelines were developed in a study that evaluated PA and exercise for health promotion in older adults and provided various strategies based on intended outcomes for lifestyle integration [34]. PA volume, intensity, and modality-specific adaptations should be considered during the prescription of PA/exercise for health. Hence, individualized PA/exercise programs would be desirable based on the intended outcomes. In this regard, our study is meaningful in that it summarizes the amount of PA/exercise in older Korean adults, which may be reflected in the ICFSR consensus guidelines. A standard approach in IPAQ-SF is an analytical method based on metabolic equivalents (MET) [35]. However, a systematic review revealed that the IPAQ-SF has a low validity, although correlations of IPAQ-SF score have been observed with amount of vigorous activity and walking [36, 37]. A previous study on a similar cohort that used MET-min per week to determine the relationship between HGS and total PA amount yielded results that are comparable to ours [31]. However, in routine clinical practice, exercise is prescribed in terms of its type and intensity rather than according to MET, and we believe our use of these variables to be a strength of our study [36].

Other strengths of this study are that it includes a large representative population with weighted data that reflects nationwide prevalence estimates, uses recent criteria for sarcopenia, and categorizes PA based on intensity, duration, and frequency [21, 22]. In addition, instead of constructing an exercise program to analyze its effectiveness, we classified exercise patterns based on the validity of the IPAQ-SF in Koreans. Nevertheless, this study has several limitations. First, this was a cross-sectional study. Thus, we were not able to identify causal relationships. Furthermore, we cannot rule out reverse causation: good muscle mass and muscle strength may lead to increased PA in older adults. Although such an interpretation cannot be excluded, the following should be considered: Aerobic exercise induces ATP production in the mitochondria in skeletal muscles and improves aerobic capacity and muscle protein synthesis [38]. In addition, aerobic exercise influences mRNA expression of myostatin and autophagy protein [39, 40]. Resistance exercise is an important strategy for preventing muscle atrophy and increasing muscle strength and mass [11, 12]. Given the limitations of our study and the importance of preventing sarcopenia, further studies are warranted to conclude that the intensity of exercise impacts muscle mass and strength. Second, obtaining both SMI and HGS data from a single cohort would provide better results and enable more complex analyses. However, since the KNHANES was conducted for multidisciplinary purposes, two tests with a similar purpose might not be performed concurrently in a cohort. In addition, comparison of different cohorts provided insight into relevant clinical objectives. Nevertheless, using different definitions of sarcopenia based on muscle strength and mass makes the interpretation of the true impact of PA difficult, and additional research with trend analysis is needed to address these issues. Third, as all information was obtained through self-reported health surveys, there is the potential for recall or acquiescence bias, which could lead to misclassification. Fourth, the relationship between PA amount and sarcopenia might have been estimated incorrectly in our analyses, which were predominantly based on PA intensity. Similar results may be obtained in retrospective studies; therefore, confirmation of our findings through prospective studies is warranted. Fifth, there may be a potential for selection bias or data missing not at random, since all data on missing exposures and outcomes were removed from the analyses. The data on muscle mass and strength of some older adults might have been missing because they were too old and physically weak to go out. To address these issues, missing demographics were ascertained. The mean age of the 2,942 missing individuals was 79 years, and the mean age of the study participants was 69 years, with no significant differences between the two. As a result, we believe these concerns to be minor.

## Conclusions

PA intensity was positively correlated with SMI and HGS in men and women aged ≥ 60 years. Logistic regression analysis revealed a strong relationship between PA intensity and SMI and HGS, suggesting that high intensity PA may have protective effects against sarcopenia. In men, the effects of PA are clearly observed in muscle mass and strength. In contrast, in women, the effects of PA are reflected in HGS rather than SMI, and further studies are warranted to investigate this difference.

## Data Availability

The datasets used and/or analyzed during the current study are available from the Korea National Health & Nutrition Examination Survey (KNHANES) official website. (https://knhanes.kdca.go.kr/knhanes/eng/index.do).

## Abbreviations

ASM: Appendicular skeletal muscle mass
AWGS: Asian Working Group for Sarcopenia
BMI: Body mass index
CI: Confidence interval
DXA: Dual-energy X-ray absorptiometry
HGS: Hand grip strength
ICFSR: International Exercise Recommendations in Older Adults
IPAQ-SF: International Physical Activity Questionnaire-Short Form
KNHANES: Korea National Health and Nutrition Examination Surveys
OR: Odds ratio
MET: Metabolic equivalent
PA: Physical activity
SE: Standard error
SMI: Skeletal muscle index
